# Transcriptional and biochemical analyses of *Planomicrobium* strain AX6 from Qinghai-Tibetan Plateau, China, reveal hydrogen peroxide scavenging potential

**DOI:** 10.1186/s12866-022-02677-w

**Published:** 2022-11-05

**Authors:** Ali Bahadur, Ting Li, Wasim Sajjad, Fahad Nasir, Muhammad Amir Zia, Minghui Wu, Gaosen Zhang, Guangxiu Liu, Tuo Chen, Wei Zhang

**Affiliations:** 1 State Key Laboratory of Cryospheric Science, Northwest Institute of Eco-Environment and Resources, Chinese Academy of Sciences, Lanzhou, 730000 China; 2 Key Laboratory of Extreme Environmental Microbial Resources and Engineering, Gansu Province, Lanzhou, 730000 China; 3 School of Ecology and Environmental Science, Yunnan University, Kunming, 650091 China; 4 Key Laboratory of Mollisols Agroecology, Northeast Institute of Geography and Agroecology, Chinese Academy of Sciences (CAS), Changchun, 130102 Jilin Province China; 5 National Institute for Genomics and Advanced Biotechnology (NIGAB), National Agriculture Research Center (NARC), Islamabad, Pakistan; 6 Key Laboratory of Desert and Desertification, Northwest Institute of Eco-Environment and Resources, Chinese Academy of Sciences, Lanzhou, 730000 China; 7 Key Laboratory of Extreme Environmental Microbial Resources and Engineering, Lanzhou, 730000 Gansu Province China

**Keywords:** Biochemical analysis, Hydrogen peroxide, *Planomicrobium* strain AX6, Qinghai-Tibetan Plateau, Transcriptional profiling

## Abstract

**Background:**

The bacterial mechanisms responsible for hydrogen peroxide (H_2_O_2_) scavenging have been well-reported, yet little is known about how bacteria isolated from cold-environments respond to H_2_O_2_ stress. Therefore, we investigated the transcriptional profiling of the *Planomicrobium* strain AX6 strain isolated from the cold-desert ecosystem in the Qaidam Basin, Qinghai-Tibet Plateau, China, in response to H_2_O_2_ stress aiming to uncover the molecular mechanisms associated with H_2_O_2_ scavenging potential.

**Methods:**

We investigated the H_2_O_2_-scavenging potential of the bacterial *Planomicrobium* strain AX6 isolated from the cold-desert ecosystem in the Qaidam Basin, Qinghai-Tibet Plateau, China. Furthermore, we used high-throughput RNA-sequencing to unravel the molecular aspects associated with the H_2_O_2_ scavenging potential of the *Planomicrobium* strain AX6 isolate.

**Results:**

In total, 3,427 differentially expressed genes (DEGs) were identified in *Planomicrobium* strain AX6 isolate in response to 4 h of H_2_O_2_ (1.5 mM) exposure. Besides, Kyoto Encyclopedia of Genes and Genomes pathway and Gene Ontology analyses revealed the down- and/or up-regulated pathways following H_2_O_2_ treatment. Our study not only identified the H_2_O_2_ scavenging capability of the strain nevertheless also a range of mechanisms to cope with the toxic effect of H_2_O_2_ through genes involved in oxidative stress response. Compared to control, several genes coding for antioxidant proteins, including glutathione peroxidase (GSH-Px), Coproporphyrinogen III oxidase, and superoxide dismutase (SOD), were relatively up-regulated in *Planomicrobium* strain AX6, when exposed to H_2_O_2_.

**Conclusions:**

Overall, the results suggest that the up-regulated genes responsible for antioxidant defense pathways serve as essential regulatory mechanisms for removing H_2_O_2_ in *Planomicrobium* strain AX6. The DEGs identified here could provide a competitive advantage for the existence of *Planomicrobium* strain AX6 in H_2_O_2_-polluted environments.

**Supplementary Information:**

The online version contains supplementary material available at 10.1186/s12866-022-02677-w.

## Background

One of the critical challenges for living organisms is to overcome the oxidative stress caused by reactive oxygen species (ROS), including hydrogen peroxide (H_2_O_2_), hydroxyl radicals (^˙^OH), and superoxide anions (O_2_^˙¯^), that cause damage to virtually all macromolecules of the cell [1, 2]. For instance, the higher concentration of ROS could damage cellular proteins [3], lipids [4], and DNA [5], leading to several growth and metabolic defects, thereby causing cell death. To counteract these lethal effects, living organisms have developed different mechanisms at the genetic, molecular, and biochemical levels [1, 6]. For instance, when bacteria were exposed to the mM levels of H_2_O_2_, they produced catalase (CAT) to scavenge H_2_O_2_, while mutants with impaired CAT activity lost their H_2_O_2_ scavenging capability [7]. Furthermore, bacteria are equipped with enzymatic antioxidant defense systems to cope with higher or lower concentrations of H_2_O_2_ [8, 9]. To avoid the lethal effects of H_2_O_2_, effective natural antioxidants seem a vital target to be discovered. Therefore, identifying new bacterial taxa that could produce potent antioxidants for removing H_2_O_2_ remained the main aim of this study.

Since specific bacteria have preferred several vital ecological processes and sustaining life for decades [10]; therefore, understanding the bacterial ecosystem is gaining special attention. However, a major portion of these microbes and their ecological functions in desert ecosystems are yet to be explored. Coping with oxidative stress by activating antioxidant defense systems is among the vital functions of microbes [1, 11, 12]. The Qaidam Basin of the Qinghai-Tibet Plateau is a cold hyper-arid desert at an altitude of more than 4,500 m [13]. Diverse bacterial communities dominate these high-elevation cold-desert ecosystems, frequently subjected to stressful conditions like low atmospheric oxygen content, excessive ultraviolet (UV) radiation, low air temperatures, and low nutrient availability [13–15]. These extreme environmental conditions serve as a potential source of the genomic pool and are considered suitable regions for antioxidant-producing bacteria.

To cope with H_2_O_2_ stress, bacteria use multifaceted defense mechanisms by producing antioxidant enzymes, including superoxide dismutase (SOD), glutathione peroxidase (GSH-Px), and CAT [7, 16–18]. Among bacteria, *Escherichia coli* was the first identified model of enzyme regulator, and various defense-related genes have been activated for H_2_O_2_ scavenging, thereby providing the best protective system against oxidative stress [2, 19, 20]. On the other hand, the Gram-positive bacterial strain *Deinococcus radiodurans* utilizes enzymatic antioxidants to sense oxidative stress and scavenge ROS [21, 22]. The H_2_O_2_ stress could cause significant damage and inactivate the functions of DNA, lipids, and proteins, leading to several metabolic deficiencies and mutations [2, 8, 23]. Previously, it was reported that the process of H_2_O_2_-mediated degradation of cellular machinery was more complex. Over the last decade, researchers have recognized enzymatic defense mechanisms in bacteria that can scavenge H_2_O_2_ in vitro, while in vivo research displays a weaker response to H_2_O_2_ [2, 8, 24]. The lack of knowledge about cellular resistance against H_2_O_2_ and the resultant products of cellular metabolism hinders to development advance cellular approaches for combating toxic effects. Hence, a better understanding of the molecular events that respond to H_2_O_2_ toxicity might improve bacterial adaptation and optimization [7, 16].

In the last decade, rapid advancements in next-generation sequencing techniques and bioinformatics have made the cost of sequencing reasonable. For instance, high-throughput RNA-sequencing (RNA-Seq) has been broadly used to analyze transcriptional changes across the whole genome [25, 26]. In various bacterial species for which genomic information is absent, transcriptional profiling seems a powerful technique for studying the H_2_O_2_ adaptation mechanism and other biological characteristics [7, 8, 27]. In different species of bacteria, several H_2_O_2_-induced genes have been recognized, and the expression patterns and the function of specific H_2_O_2_-induced genes have been studied [28–30]. This suggests that the biochemical and physiological responses are governed by a network of DEGs that controls complex molecular mechanisms in bacteria to adapt against H_2_O_2_ stress.

The genus *Planomicrobium* was originally described by Yoon et al. [31]. There were six valid speciesin the genus *Planomicrobium*until recently: *Planomicrobium psychrophilum*, *P. chinense* [32], *P. koreense*, *P. mcmeekinii*, *P. okeanokoites* [31], and *P. alkanoclasticum* [32, 33]. The known characteristics of the genus *Planomicrobium* are that the species are Gram-positive to Gram-variable, aerobic, yellow-to orange-pigmented, and non-spore-forming, respectively. *Planomicrobium* species contained DNA G and C in the range 35–47 mol% [34]. In this study, the *Planomicrobium*-AX6 strain was isolated from the cold-desert soil in the Qaidam Basin, Qinghai-Tibetan Plateau, China which elucidated a strong antioxidant activity. The H_2_O_2_ scavenging mechanisms of *Planomicrobium* are rarely explored. Therefore, in this study, we carried out the transcriptional profiling of the *Planomicrobium* strain AX6 intending to unravel the molecular aspects associated with H_2_O_2_ scavenging potential in cold-desert ecosystems. As per our knowledge, this is the first study that presents the transcriptional analyses of any strain belonging to *Planomicrobium* in response to H_2_O_2_ stress.

## Materials and Methods

### Preparation and molecular identification of bacterial strain

The bacterial *Planomicrobium* strain AX6 was isolated from the desert sand soils (0–10 cm) in the Qaidam Basin, Qinghai-Tibet Plateau, China (37.088°N, 95.427421°E). The medium used for isolation was composed of (g/L): yeast extract, 5; peptone,10; sodium acetate, 1; ammonium nitrate, 0.2; sodium citrate, 0.5 and agar, 15; having a pH of 7.5, and incubated for 96 h at 20 °C, and further preserved in glycerol (20%, v/v) at -80 °C [35] for further processing. Readers are referred to Ting et al. [36] with regard to detailed molecular identification of this bacterial strain.

### H_2_O_2_ scavenging potential

*Escherichia coli* JM109 (strain number C1310) purchased from Solarbio Company has a low tolerance to oxidizing substances [37] and thus was used as a negative control. While *Deinococcus radiodurans* (strain number 1.3828) purchased from China Micro Ordinary Microbiology Center (CGMCC) was used as a positive control. This bacterial isolate thrives in desert ecosystems due to ultra-high radiation resistance and antioxidant capabilities [37, 38]. The details pertaining to the respective bacterial strains are shown in supplementary material Table S1. All these bacterial isolates were exposed to two different H_2_O_2_ concentrations (1.5 and 3 mM) for 4 h.

The effect of H_2_O_2_ on *Planomicrobium* strain AX6 growth was studied as follows: 100 μL inoculum of *Planomicrobium* strain AX6 in their exponential growth stage (OD_600_ = 0.6) was inoculated in 50 mL Luria Broth (LB) medium containing 0, 0.5, 1.0, 1.5, 2.0, and 2.5 mM H_2_O_2_ and incubated at 28 °C for 103 h at the speed of 200 rpm/min. The bacterial cell concentration was estimated using the absorbance of a spectrophotometer (600 nm). All these experiments were done in triplicates.

### ROS scavenging potential assay

The scavenging ability of three different ROS agents by *Planomicrobium* strain AX6 was evaluated. For this purpose, the bacterial inoculums were mixed with phosphate buffer solution (PBS) and further suspended with 2,2-diphenyl-1-picrylhdrazyl (DPPH)•ethanol according to the method used by Lee et al. [39], and DPPH scavenging was estimated. Moreover, the bacterial inoculum was mixed with Tris–HCl buffer, and the method used by Wang et al. [40] was adopted for studying the O_2_^¯^ scavenging potential of the strain. For determining the hydroxyl radical (˙OH) scavenging potential, the bacterial inoculum was mixed with sodium phosphate buffer according to the method and estimation used by Das and Goyal. [41]. The experiments mentioned above were repeated three times, and mean results were reported in this study.

### Estimation of SOD, CAT, and GSH-Px

For enzymatic analyses, the SOD was extracted using the pyrogallol auto-oxidation method [42]. Furthermore, CAT content assay was determined by spectrophotometry, hydrogen oxide absorbance change value to characterize, the procedure was modified from Nakayama et al. [43]. The activity of GSH-Px was studied by using the colorimetric method with the help of Leagene kit (Beijing Leagene Biotech Co., Ltd., China).

### RNA-sequencing

The extraction of total RNA from 0 and 1.5 mM H_2_O_2_ concentrations was performed using the TRIzol® Reagent RNA Purification Kit for bacteria (Invitrogen) according to the manufacturer’s instructions. A Nanodrop 2000 spectrophotometer evaluated the integrity and the quality of the extracted total RNA. All RNA samples were DNAse treated using the Ribo-Zero Magnetic kit to eliminate every contaminating genomic rRNA. The experiments were performed in triplicate for each stress treatment. RNA sequencing was done on the Illumina HiSeq4000 according to the manufacturer’s protocol (Illumina Inc., Majorbio, China). Briefly, the quality and the integrity of the total RNA were evaluated using the Agilent 2100 Bioanalyzer. RNA with an RNA Integrity Number (RIN) of 7.0 or higher was used for sequencing library preparation and processing. Sequencing libraries were adjusted using the TruSeq Stranded Specific mRNA Library sample Prep Kit as per the manufacturer’s guidelines (Illumina). Cluster generation was done according to the manufacturer’s references for onboard clustering (HiSeq 4000 PE Cluster Kit, Illumina). Next, sequencing (2 × 150 bp) was performed using Illumina Hiseq 300 platform.

### Bioinformatics analysis

The quality of FASTQ files was evaluated using FASTQC v. 0.11.8 [44]. Generally, the default parameters were used to align the reads to the *Planomicrobium* strain AX6 genomes to assess contamination and further aligned to the bacterial genomes assembly using Bowtie2(http://www.bowtie-bio.sourceforge.net/bowtie2/manual). The transcript abundances were processed using RSeQC-2.6.3 (http://rseqc.sourceforge.net/), having strand-specific read counting. Data were normalized, and the DEGs were quantified using the edgeR (http://www.bioconductor.org/packages/2.12/bioc/html/edgeR.html) by applying criteria of having corrected *P*-value or FDR < 0.05 and fold change log^2^FC ≤—1 or ≥ 1 [45–47]. Differential gene enrichment analyses for Gene Ontology (GO) (https://github.com/tanghaibao/GOatools) and Kyoto Encyclopedia of Genes and Genomes (KEGG) metabolic pathways (http://www.genome.jp/kegg/) were performed using Goatools and KOBAS function [48, 49], respectively, and the statistical method used was Fisher’s exact test. The dominant metabolic pathways were analyzed through KEGG enrichment analysis having FDR < 0.05 [50]. The fitting growth curves of *Planomicrobium* strain AX6 were drawn with R-package (*plyr*, *ggplot2*) v 3.6. The Principal Component Analysis (PCA) was analyzed to find outliers and discriminate sample clusters with high similarities. The scatter plot represents genes' expression level (FPKM value) in the control and treatment samples [47]. The heatmap was generated to find genes with significant differences by clustering their expression patterns using hcluster (complete algorithm). All analyses were carried out via R-Vagan v. 6.3.

## Results

### Identification and growth adaptation attributes of Planomicrobium strain AX6 against H_2_O_2_ stress

*Planomicrobium* strain AX6 was identified at the molecular level by Ting et al. [36] using *16 s rRNA* sequencing. More than 80% of the *Planomicrobium* strain AX6 showed resistance to 0.02—1.0 mM H_2_O_2_, and there was no significant variation in the survival rate. Though, after 1.5 mM H_2_O_2,_ it reduced to 56% and showed a substantial change in survival rate, then to 45% and 27% on 2.0 mM and 3.0 mM, respectively (Fig. S1A). *Planomicrobium* strain AX6 growth was decreased (cultural density at OD_600_) after 103 h in 2.0 and 2.5 mM H_2_O_2_ treatment (Fig. S1B). We selected a concentration of 1.5 mM H_2_O_2_ for further study. As shown in Figure S1B, the growth rate was significantly inhibited by 1.5 mM of H_2_O_2_ between 10 and 49 h of treatment, demonstrating that *Planomicrobium* strain AX6 is directly involved in H_2_O_2_ scavenging.

### Bacterial comparison for H_2_O_2_ scavenging ability

The DPPH free radical scavenging rate is usually used to determine the antioxidant capacity. Therefore, the scavenging rates of DPPH, ^˙^OH, and O_2_^˙¯^ were measured to characterize the antioxidant characteristics of *Planomicrobium* strain AX6 (Table S1). In addition, *Planomicrobium* strain AX6 to remove H_2_O_2_ with different concentrations (0, 1.5, and 3 mM) was verified. The DPPH, O_2_^˙¯^, and ^˙^OH scavenging potentials of *Planomicrobium* strain AX6 were significantly higher (16.6, 18.3, and 36.4%) than the negative control (5.0, 9.5, and 6.1%) (*P* < 0.05) while lower than the positive control (20.7, 21.8, 53.0%) when treated with 1.5 mM of H_2_O_2_ (Table S1). These results confirmed the antioxidant potential of *Planomicrobium* strain AX6.

### Estimation of SOD, CAT, and GSH-Px

**Fig. 1:**
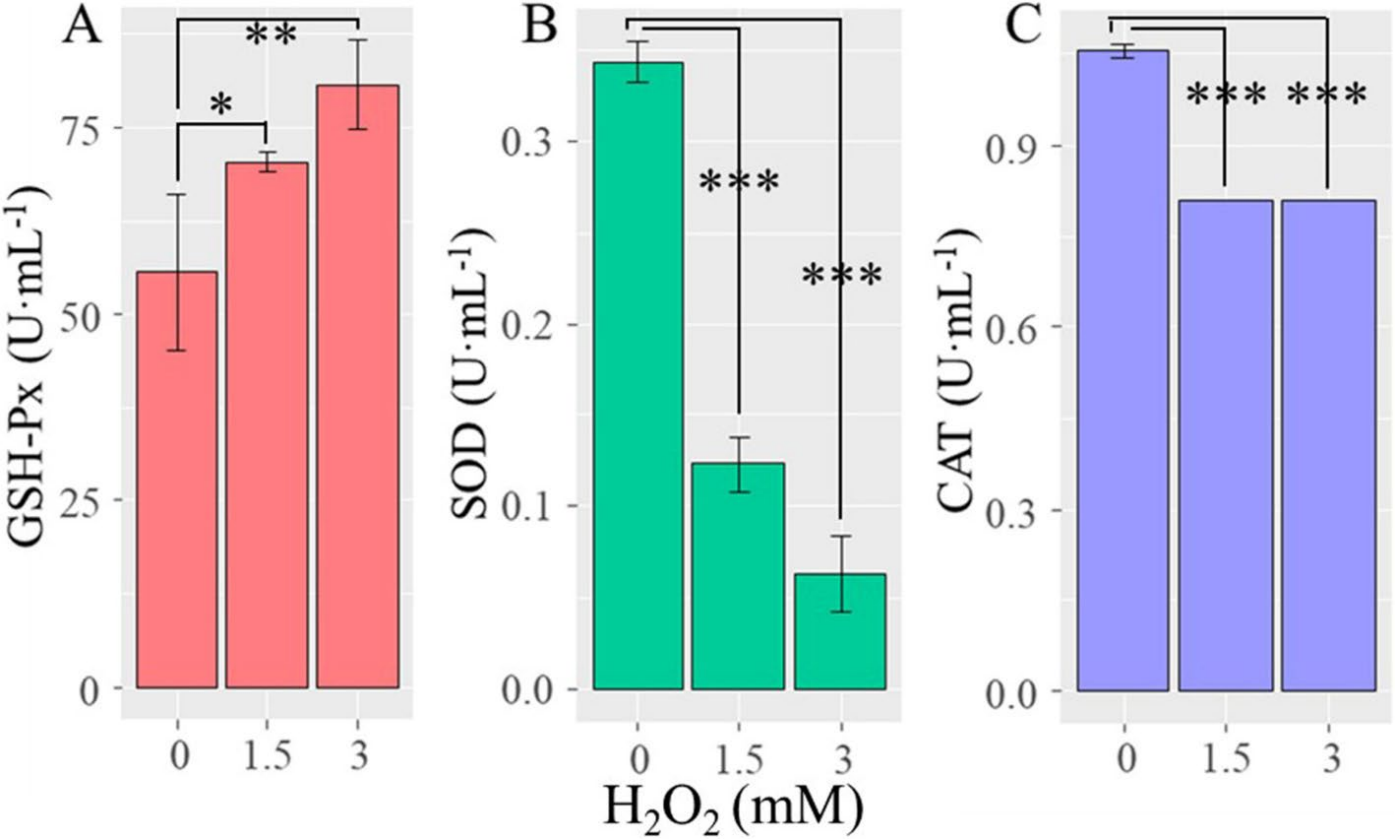
Antioxidant enzyme activity of *Planomicrobium*-AX6 exposed to H_2_O_2_ treatments. **A** glutathione peroxidase (GSH-Px), **B** superoxide dismutase (SOD), and **(C)** catalase (CAT) production rate when exposed to H_2_O_2_ (0, 1.5, and 3 mM) for 4 h. Where asterisks indicate significant differences. ***P < 0.001; **P < 0.01; *P < 0.05

As illustrated in Fig. 1A, upon 1.5 and 3 mM of H_2_O_2_ treatment, GSH-Px activity was significantly higher than the control (0 mM H_2_O_2_) (*P* < 0.05). With the increase in H_2_O_2_ levels, the SOD and CAT activity of *Planomicrobium* strain AX6 decreased significantly (Fig. 1B and C). This suggests that SOD is likely to be responsible for the conversion of O_2_^˙¯^ into H_2_O_2_, where the oxidation environment dominated by H_2_O_2_ inhibits the process of SOD [51], which as a result, might decrease the SOD activity.

### Transcriptional profiling of the Planomicrobium strain AX6 in response to H_2_O_2_ stress

To gain insights into genes and key metabolic pathways responsible for H_2_O_2_ regulation by *Planomicrobium* strain AX6, their transcriptional profiling was conducted in response to H_2_O_2_ stress. The Illumina/Truseq™ RNA platform generated two libraries of bacterial included (AX6) and control (CK) groups comprising 22,260,393 and 23,723,531 raw reads, respectively (Table S2). The length of a single read was 200 bp. After removing the short reads, contaminated reads, and adaptor sequences from *Planomicrobium* strain AX6 and CK group libraries, a total of clean reads comprising 21,936,575 and 23,271,179, having Q20 values 98.89 and 98.75%, respectively, and Q30 values were 96.59 and 96.17%, respectively. Moreover, the GC content values were 44.03 and 45.66%, while 98.90 and 98.62% of transcripts have been mapped, respectively (Table S2). Furthermore, 770,388 transcripts were composed of high-quality reads using Glimmer 3.02 software. The average length was 832 bp. Scaffold N50 and N90 values were 55, 491, 2, and 59,610.

### Gene expression pattern and correlation between control and H_2_O_>2_ stress

**Fig. 2:**
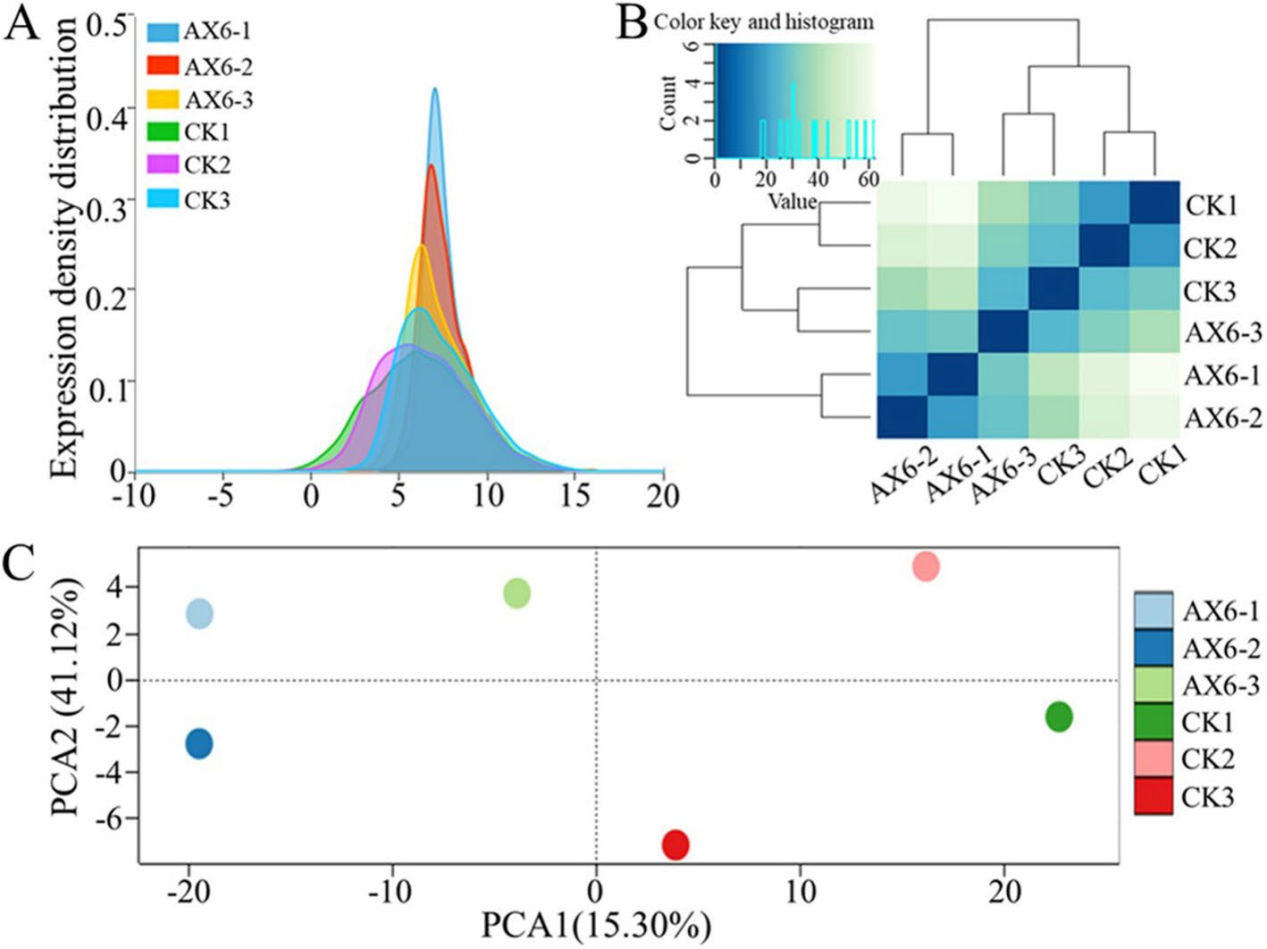
Density curve, hierarchical cluster heatmap, and Principal component analysis (PCA) of *Planomicrobium*-AX6 transcriptome data, exposed to hydrogen peroxide for 4 h under H_2_O_2_ (1.5 mM). **A** Density curve. The probability density distribution of the expression of all genes was log.^2^FPKM, the peak of the density curve represents the region where the gene expression of the entire sample is most concentrated. Each color in the figure represents a sample, and the sum of all probabilities is 1. **B** Hierarchical cluster heatmap. The correlation analysis between samples, the closer the correlation coefficient is to 1, the higher the similarity between samples. According to the quantitative results of FPKM, I calculated the correlation between all the samples. **C** Principal component analysis (PCA). The PCA demonstrates of expressed genes (FPKM ≥ 0.01) a clear separation (Axis 1) of AX6 vs.CK treatment. CK designate RNA sample replicates obtained from *Planomicrobium*-AX6 without H_2_O_2_ (1.5 mM) for 4 h, whereas AX6 is RNA sample replicates obtained from *Planomicrobium*-AX6 with H_2_O_2_ (1.5 mM) for 4 h. CK1-3 control; AX1-3 treatments, *n* = 3

According to RSEM software, the log2FPKM distribution between the control and H_2_O_2_ suggested the highly expressed genes under stress conditions compared to the control (Fig. 2A, B), which exhibited a pattern consistent with the main biological replicates that meet the expectations of the experimental design. The statistical results of each sample's correlation are shown in Fig. 2B. The columns correspond to the FPKM ≥ 0.01 of each gene, and the rows correspond to the six samples. Similarly, the scores matrix delivered sample scores for gene expression patterns, labeling the profiling of gene expression distribution between control and stress treatment. The biological triplicate samples of each group were clustered together. The control and stress treatment groups were plotted distinctly along the PC1 direction (Fig. 2C).

### Identification of DEGs in Planomicrobium strain AX6 exposed to H_2_O_2_

**Fig. 3:**
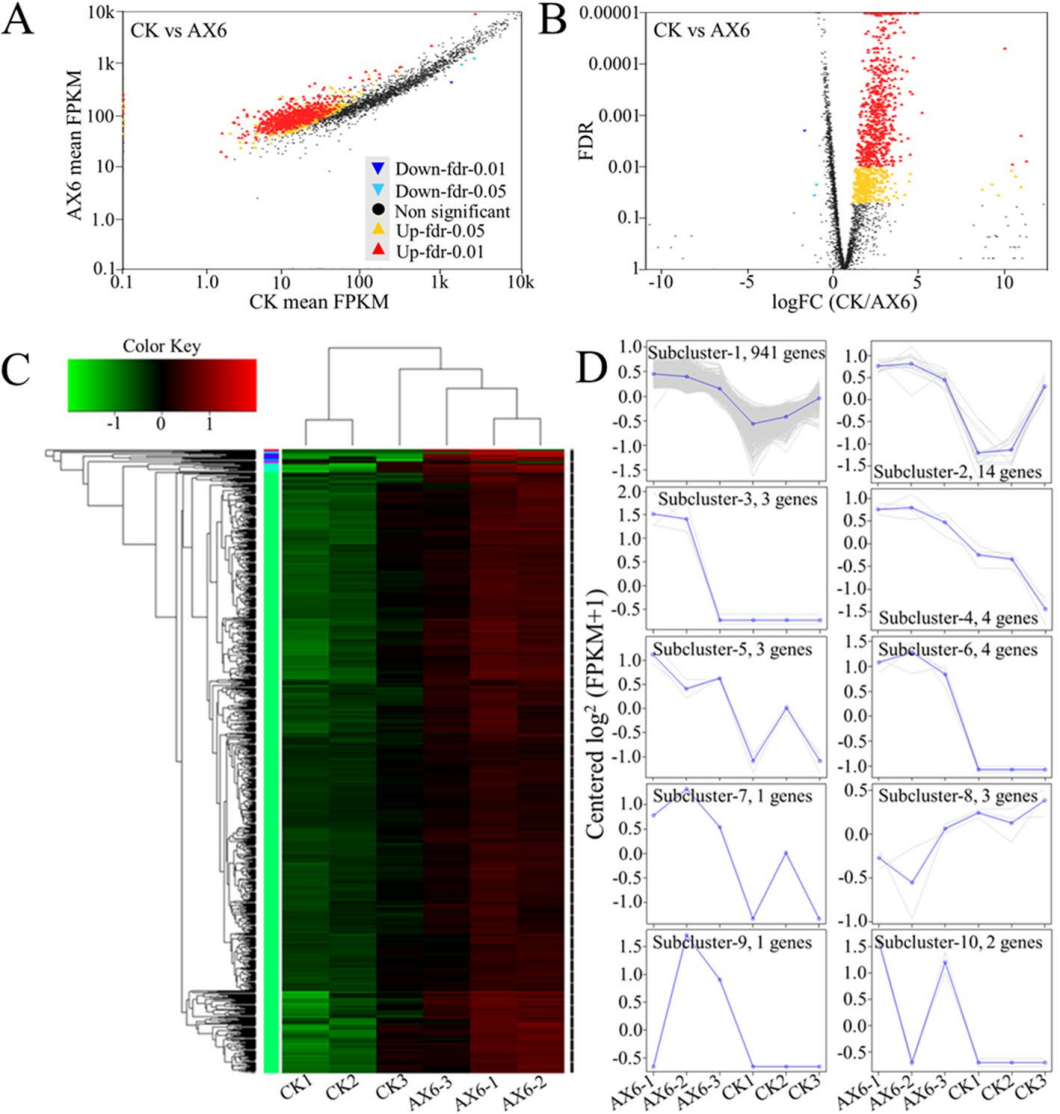
Heatmap, scatter, and volcano plot of DEGs of *Planomicrobium*-AX6 transcriptome data, exposed to hydrogen peroxide for 4 h under H_2_O_2_ (1.5 mM). **A** Scatter plot. The horizontal and vertical coordinates have logarithmic values, indicate the expression levels (FPKM value) of genes in the AX6 and CK. **B** Volcano plot. Each colored point in the volcano plot represents a single gene. Plotted along the x-axis is the log2 fold change of each gene. The y-axis represents the negative logarithm of the corresponding *P*-value of that gene. **C** Heatmap. The scale bar shows the z-score for a differentially expressed gene. The color represents the amount of gene expression in the group of samples (log.^10^ FPKM), referring to the color key at the upper left. **D** Line plot. The sub-cluster of the heatmap represents a gene, and the blue line represents the average expression of all genes in the sub-cluster. CK designate RNA sample replicates obtained from *Planomicrobium*-AX6 without H_2_O_2_ (1.5 mM) for 4 h, whereas AX6 is RNA sample replicates obtained from *Planomicrobium*-AX6 with H_2_O_2_ (1.5 mM) for 4 h. CK1-3 control; AX1-3 treatments, *n* = 3

To find the variation in transcriptional profiling of *Planomicrobium* strain AX6 exposed to H_2_O_2_, the gene expression changes of both control and stress treatments were compared. In this study,

statistical comparisons employed (*P*-value) indicating significant differences between *Planomicrobium* strain AX6 and CK control, the DEGs were regarding the two criteria, the average fold change was ≤ 2 having *P*-value > 0.01 and FDR ≤ 0.001 (For instance of AX6 vs. CK, Fig. 3A and B). A total of 3,427 significant DEGs were identified. Among them, the number of up-regulated transcripts (2833) was higher compared to down-regulated (594) at the 4 h under H_2_O_2_ (*P* > 0.05) (Table 1; Fig. 3A and B), there was rich variation in response to H_2_O_2_. We found that the expressions differed for analyzed genes, with significant differences between control and stress treatment. For transcriptional profiling of *Planomicrobium* strain AX6 in response to H_2_O_2_ stress, the transcription profiles of 6 samples were analyzed using a clustering algorithm and tree view (Fig. 3C). They exhibited different profiles at the time points of the pairing between the two groups and each group had a different sample-specific profile. Commonly, similar transcription patterns were identified across control (CK1, CK2, CK3), while the up-regulated genes were reported in stress treatment (AX6-1, AX6-2, AX6-3), respectively. The samples of CK3 and AX6-3 shared similar transcription patterns (Fig. 3C). In addition to this, 976 stress-responsive DEGs were found that were further divided into 10 sub-clusters. The average profile for each group in each sub-cluster and the existent profile variances between the *Planomicrobium* strain AX6 and the control group are shown in Fig. 3D. For instance, in sub-clusters 1, 2, and 4 to 7, the gene expression level under stress was up-regulated, while it was down-regulated in CK. In sub-cluster 8, the gene expression was down-regulated upon stress, whereas it was up-regulated in CK. Similarly, in sub-cluster 9, the gene expression was down-regulated in AX6-1 and up-regulated in AX6-2 and AX6-3, remaining stable from CK1 to CK3, respectively. Finally, in sub-cluster 10, the gene expression in AX6-2 was comparable with the control (Fig. 3D).

**Table 1:** The number of DEGs in AX6 when exposed to H_2_O_2_ (1.5 mM) for 4 h

Differently expressed genes	Number of DEGs^a^
DEGs Up-regulation	2833
DEGs Down-regulation	594
No change	81
Total	3508

^a ^Limma was used to identify DEGs (FDR < 0.05)

### Functional annotation

**Fig. 4:**
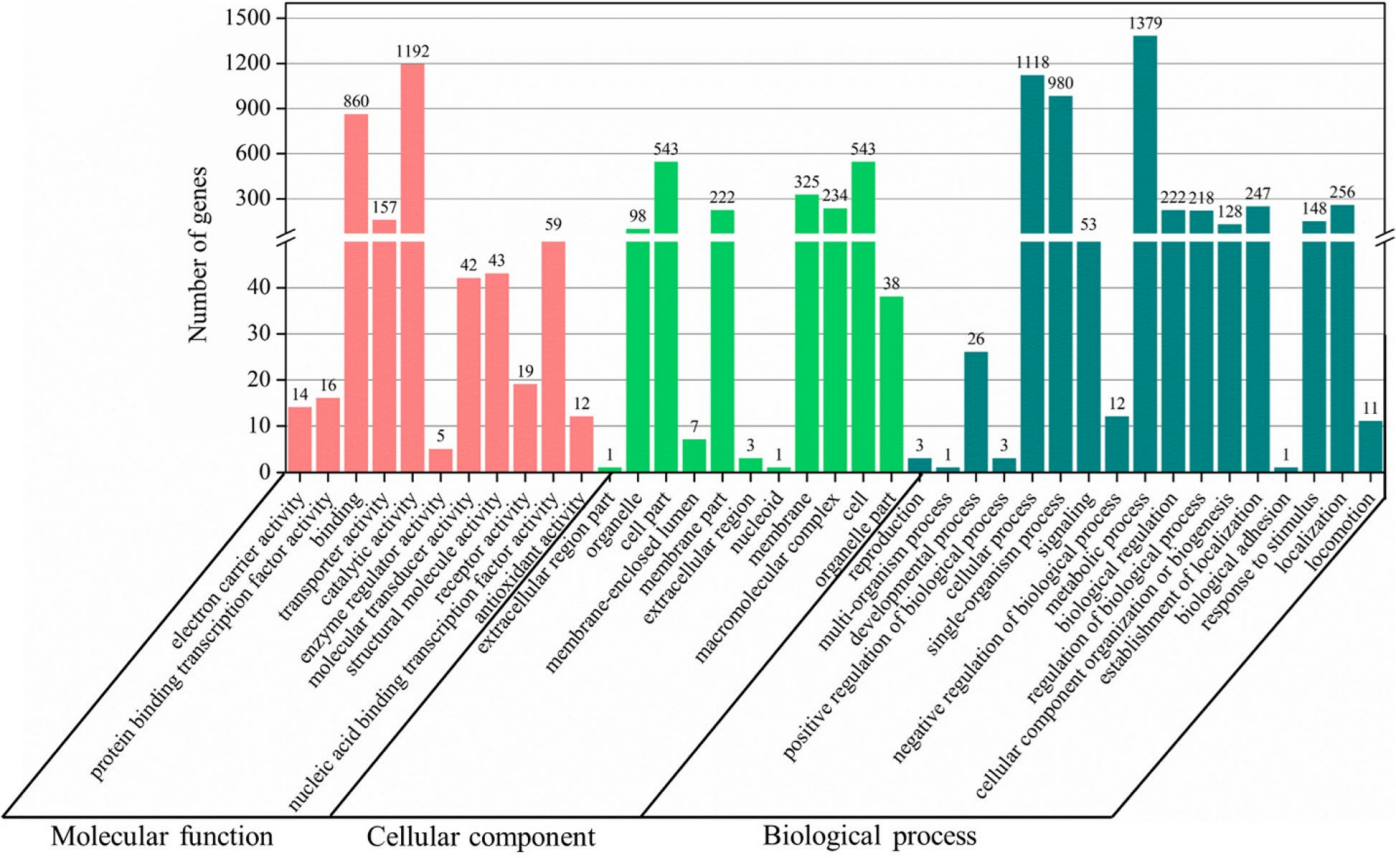
Gene ontology (GO) classification of DEGs for *Planomicrobium*-AX6 between the H_2_O_2_ (1.5 mM) for 4 h and the control samples without H_2_O_2_ (1.5 mM) for 4 h. The red bars represent up-regulated genes, the blue bars represent down-regulated genes. The lower axes represents the number of genes annotated to a GO term, and the upper axes represents the ratio of the number of genes annotated to a GO term to the total number of all GO-annotated genes

The functions of all the DEGs recognized in this work were classified by GO assignments (http://www.geneontology.org/). A total of 3,427 DEGs were annotated into 2,345 functional groups in the two categories in the three basic ontologies as cellular component, molecular function, and biological process. The study covered 39 functional groups comprising 11 for cellular components and molecular function and 17 for biological processes. Metabolic process and catalytic activity were the two largest groups, and the smallest group were extracellular region part, nucleoid, and biological adhesion, with only one GO enrichment predicted for each group (Fig. 4).

Clusters of Orthologous Groups (COG) assignments were also used to estimate and classify possible functions of the DEGs. Based on sequence homology, DEGs could be characterized into 21 COG groups. Metabolism group and amino acid transport with 173 COG were the most prominent groups, followed by an unknown function (161), general function prediction only (143), ribosomal structure, translation, and biogenesis (136), and metabolism group and inorganic ion transport (123) (Fig. S2). The chromatin structure and dynamics groups were the smallest, with only one COG.

### ntioxidant genes regulation in Planomicrobium strain AX6 exposed to H_2_O_2_

The DEGs reported in *Planomicrobium* strain AX6 in response to H_2_O_2_ were ascribed to many genes encoding antioxidant enzymes. In addition, KEGG pathways enrichment analysis of the identified DEGs recognized many metabolic pathways that seemed to be potentially affected by the addition of H_2_O_2_ to *Planomicrobium* strain AX6. For instance, our results indicated that the defensive line against H_2_O_2_ in *Planomicrobium* strain AX6 comprises GSH-Px, Coproporphyrinogen III oxidase, SOD, and CAT (Table 2). The gene encoding GSH-Px (*Gpx*, orf03455_E1.11.1.9) was up-regulated by 10.15-fold in response to H_2_O_2_, while the GSH-Px gene (orf02189_ E1.11.1.9) was down-regulated 1.21-fold (Table 2). Genome sequence analysis showed two *Gpx* genes present in *Planomicrobium* strain AX6. The Gpx systems are the leading and highly studied enzymatic system that eliminates H_2_O_2_ in different bacteria. The Coproporphyrinogen III oxidase genes (orf00260_ *hemN*, *hemZ*, orf03367_ *hemL*, and orf00170_ *hemL*) were up-regulated by 5.31-, 2.34-, and 1.19-fold in H_2_O_2_ treatment samples (Table 2). The SOD gene (orf00300_ *SOD2*) was down-regulated by 1.34-fold, and other SOD genes (orf03456_*guaB* and orf03437_*cysK*) were up-regulated by 1.02- and 1.17-fold in H_2_O_2_, respectively. SOD catalyzes the dis-mutation of superoxide into H_2_O_2_ and O_2_, and therefore *orf03456* and *orf03437* might be vital antioxidant defenders in *Planomicrobium* strain AX6 when the cell is exposed to free radicals. Furthermore, to cope with the H_2_O_2_ stress, the gene encoding CAT (orf02500_ *katE*, *CAT*, *catB*, *srpA*, orf03059_ *K07217*, and orf01958_ *katE*, *CAT*, *catB*, *srpA*) were down-regulated by 1.21-, 1.26-, and 1.24-fold in H_2_O_2_ (Table 2). These results indicate that the capacity of oxidative stress defense in *Planomicrobium* strain AX6 with strongly decreased CAT activity was due to a decrease in the expression level of CAT genes, confirmed by CAT enzymes activity (Fig. 1). A future experiment should be conducted to determine which mechanisms *Planomicrobium* strain AX6 utilizes to down-regulate the CAT gene expression level.

**Table 2:** Bacterial antioxidant genes differently expressed when exposed to hydrogen peroxide for 4 h under H_2_O_2_ concentration (1.5 mM)

Antioxidant defense proteins	Sequence ID	KEGG gene name	Samples		Differently expressed genes^a^
			CK	AX6	
Glutathione peroxidase	orf03455 orf02189	*E1.11.1.9* *E1.11.1.9*	9.24 462.43	93.78 383.68	Up-regulated Down-regulated
Coproporphyrinogen III oxidase	orf00260 orf03367 orf00170	*hemN, hemZ* *hemL* *hemL*	16.00 85.32 1900.68	85.00 199.63 2279.58	Up-regulated Up-regulated Up-regulated
Superoxide dismutase	orf00300 orf03456 orf03437	*SOD2* *guaB* *cysK*	1455.85 224.89 179.85	1088.24 229.00 210.48	Down-regulated Up-regulated Up-regulated
Catalase	orf02500 orf03059 orf01958	*katE, CAT, catB, srpA* *K07217* *katE, CAT, catB, srpA*	1059.43 1449.28 4254.75	875.26 1154.82 3430.97	Down-regulated Down-regulated Down-regulated

^a^ The differentially expressed antioxidant genes were identified using limma (FDR < 0.05)

In addition, changes in gene expression levels analysis and DEGs between *Planomicrobium* strain AX6 and CK were primarily related to coenzyme transport and metabolism. Consequently, the DEGs involved in these functions were further described in detail (Table S3). Most of the genes were involved in thiamine metabolism, including the iron cysteine desulfurase, thiamine biosynthesis protein *ThiI*, hypothetical protein, and alkaline phosphatase up-regulated by the addition of H_2_O_2_. The genes related to cysteine desulfurase (*iscS*, *NFS1*) might be providing sulfur, which is later combined during in vivo Fe-S cluster synthesis, were observed to be up-regulated in H_2_O_2_ treatment (Table S3). Thiamine biosynthesis protein-encoding genes (such as *thiI* and *thiE*) were also up-regulated by adding H_2_O_2._

Besides, three genes encoding alkaline phosphatase (*E3.1.3.1*, *phoA*, *phoB*) were up-regulated. In addition, the environmental bacterial regulatory genes were down-regulated in H_2_O_2_ treatment. Moreover, genes responsible for oxidative stress adaptation were detected in the amino acids biosynthesis, mismatch repair, bacterial chemotaxis, fatty acid, cysteine and methionine metabolism, pentose phosphate pathway, and two-component system substitutions that might have allowed for effective functionality (Table S3).

## Discussion

The potential scavenging ability of H_2_O_2_ toxicity in the *Planomicrobium* strain AX6 recovered from desert soil in the Qaidam Basin, Qinghai-Tibetan Plateau, China, was carried out here by studying its growth and transcriptional profiling in response to H_2_O_2_ stress. This area is characterized by low temperature, oxygen level, and high radiation [13, 52], which directly cause light-induced damage to organisms, and forms an environment with high oxidation intensity [53]. In the higher H_2_O_2_ concentration, *Planomicrobium* strain AX6 showed a high survival rate coupled with a strong oxidative stress response. Previous studies have shown that different bacterial strains isolated from cold environments can adapt to the desert oxidative stress environment [12, 54–59]. In addition, *Planomicrobium* strain AX6 showed potential DPPH, O_2_^˙^, and OH, scavenging ability (Table S1), where the bacteria generate basal levels of ROS-scavenging enzymes to avoid the accumulation of endogenous H_2_O_2_ and O_2_^∙−^ [60]. The mechanism mainly comprises SOD that catalyzes the dismutation of O_2_^∙−^ to H_2_O_2_ and CAT that convert H_2_O_2_ to O_2_ and H_2_O [61, 62].

Furthermore, we studied the mechanisms that might play an essential role in the resistance of *Planomicrobium* strain AX6 against H_2_O_2_ stress using RNA sequencing. Based on RNA-seq profiling, a substantial number of genes (3,508) was reported to be distinctly expressed in the *Planomicrobium* strain AX6, signifying an extreme variation in the transcriptional profile of bacterial strain (Table 1) upon exposure to H_2_O_2_ stress. The genes are expressed differentially to elaborate on the resistant mechanism and the metabolic pathways that provide the energy needed to cope with oxidative stress. Therefore, it is reasonable to assume that might cause the weak resistance response produced against the bacterial strain or basis nutrients to be easily accessible to the bacteria to accomplish their metabolic pathways.

Transcriptional dynamics were examined in the CK and H_2_O_2_ stress-treated *Planomicrobium* strain AX6 strain (Fig. 2). The differences between the H_2_O_2_ stress treatment and CK responses were also revealed in the PCA of the transcriptional data (Fig. 2), which separated the H_2_O_2_ stress treatment and CK samples. Toxic levels of H_2_O_2_ can reach up to millimolar due to the oxidative stress caused in the host immune cells [7]. To prevent the toxicity of H_2_O_2_, the bacteria produce several scavenging enzymes to maintain intracellular H_2_O_2_ levels at the nanomolar [61]. The accumulation of high concentrations of H_2_O_2_ might cause serious damage to many cellular organelles by amending proteins and DNA. However, several genes encode antioxidant proteins/enzymes that detoxify H_2_O_2_ or repair the oxidative damage caused by oxidative stress. Furthermore, these antioxidant proteins/enzymes are vital in regulating the antioxidant defense system's expression, including the thioredoxin and catalase systems, against H_2_O_2_ and at extreme pressure with MgCl_2_ supplementation [63–65]. However, it is currently unclear what bacterial-specific genes respond to and which antioxidant enzymes regulated genes contribute to H_2_O_2_ toxicity. These are exciting concerns for future research exploring the relations between H_2_O_2_ stress and CAT defense mechanisms of cold-desert isolated bacteria.

In this study, the CAT enzymes coding genes such as (*katE*, *CAT*, *catB*, *srpA*, *K07217*, *katE*, *CAT*, *catB*, *srpA*) were slightly down-regulated, signifying that CAT might not play a key role in the scavenging of H_2_O_2_. So we were also surprised to find that relatively down-regulation of these genes had no seeming effect on H_2_O_2_ resistance in *Planomicrobium* strain AX6, despite the circumstance that CAT expression genes were invoked more strongly by CK treatment than by H_2_O_2_ [66, 67] (Fig. 1; Table 2). This might be due to the redundant nature of bacterial resistance mechanisms [7] or could be predictable since O_2_ is produced in its reaction [65]. Another possibility is that increased production of SOD likely to combine with CAT, resulting in a decreased ability of cells to remove H_2_O_2_ [51, 68]. Glutathione is a vital antioxidant, and GSH-Px catalyzes the reaction of glutathione and an extensive range of oxides [69–71]. The genes *Gpx*, orf03455_E1.11.1.9 were up-regulated while orf02189_ E1.11.1.9 was down-regulated (Table 2). We investigated that *Planomicrobium* strain AX6 has the complete KEGG pathway of glutathione metabolism and encodes as many as 2 copies of GSH-Px genes (Table 2), which shows that glutathione likely plays a key role in avoiding cellular substrates from O_2_ inactivation. In addition, coproporphyrinogen III oxidase and SOD can also protect the bacterial cells against H_2_O_2_ stress [72, 73]. *Planomicrobium* strain AX6 contains 4 genes (*hemN, hemZ, hemL1,2*) which were slightly up-regulated and thought to be involved in the coproporphyrinogen III oxidase system and 3 SOD genes in which 2 were up-regulated (*guaB and cysK*) whose products may also assist *Planomicrobium* strain AX6 in relieving H_2_O_2_ stress, while *SOD2* gene was relatively down-regulated.

We next focused on the expression patterns of thiamine metabolism-related genes, which might regulate the enzymatic activity of bacteria [29, 63]. The genes that were up-regulated include *iscS*, *NFS1* (Cysteine desulfurase), *E2.7.6.2*, *THI80*, *thiM*, *thiD* (hypothetical protein), and *thiE* (thiamin-phosphate pyrophosphorylase), and the down-regulated genes of the environmental bacterial metabolism were *glxK*, *E2.3.3.9*, *aceB*, *glcB*, *kdgK*, *glk*, *E5.1.3.3*, *galM*, and *hemL*, under *Planomicrobium* strain AX6 compared to CK condition. In addition, numerous genes associated with cysteine were up-regulated (Table S3). Cysteine plays a key role in iron transport, as a high level of cysteine is a crucial component for the biosynthesis of siderophores in iron transport [63, 74]. Cysteine is mainly derived from the conversion of other amino acids to directly acquire extracellular cysteine in the bacterial cell [75]. The expression of genes in *E. coli* is mostly controlled by S-adenosylmethionine concentrations and intracellular cysteine [76–79], signifying that methionine synthesis, cysteine, and transport genes are up-regulated by H_2_O_2_ in *Planomicrobium* strain AX6. The up-regulated genes encode permease, SAM-dependent methyltransferase, 5'-methylthioadenosine/S-adenosylhomocysteine nucleosidase, and aspartate kinase. Furthermore, chemotaxis plays a crucial role and the genes that were up-regulated include *fliNY, fliN, fliM* (Hypothetical protein), *motB* (Flagellar motor protein MotB), and *motA* (Flagellar motor protein MotA), under Planomicrobium strain AX6 compared to CK condition. However, it is not clear whether the up-regulation of these genes could protect cells against H_2_O_2_ stress. An overview of *Planomicrobium* strain AX6, when exposed to H_2_O_2_ concentration (1.5 mM) for 4 h, including antioxidant genes that avoid cell damage, was summarized in Table 2.

Overall, the findings of this study provide a clue to the biological roles and mechanism of the *Planomicrobium* strain AX6 in H_2_O_2_ scavenging. The above-mentioned antioxidant systems proposed that the bacterial strain could protect proteins from the toxicity produced during H_2_O_2_ stress. In addition to the above-annotated genes, numerous genes with unknown functions exist in the function of *Planomicrobium* strain AX6. In this study, several genes coding for antioxidant proteins, including glutathione peroxidase (GSH-Px), Coproporphyrinogen III oxidase, and superoxide dismutase (SOD), were induced in *Planomicrobium* strain AX6 in response to H_2_O_2_ when compared with the control. Taken as a whole, the findings propose that the induced transcripts responsible for antioxidant defense pathways serve as important regulatory mechanisms for scavenging H_2_O_2_ in *Planomicrobium* strain AX6. The DEGs presented in this study could provide a competitive advantage for the survival of *Planomicrobium* strain AX6 in H_2_O_2_-polluted environments.

## Conclusion

In this study, *Planomicrobium* strain AX6 has been studied for its remarkable H_2_O_2_ scavenging potential. Based on RNA-seq data, it is evident that *Planomicrobium* strain AX6 could potentially lead to severe variations in the numerous metabolic pathways involved in generating an antioxidant defense system in response to H_2_O_2_ stress, consequently reducing the toxicity of the H_2_O_2_ stress. We found many slightly up-regulated DEGs encode proteins involved in antioxidant defense like GSH-Px, Coproporphyrinogen III oxidase, SOD, and other H_2_O_2_ scavenging-related metabolisms. To our surprise, in this study, CAT was relatively down-regulated, signifying that CAT might not play a key role in the scavenging of H_2_O_2_ in *Planomicrobium* strain AX6. Collectively, the data have provided several pathways and candidate genes for future functional genomics work. This study opens new research doors to explore bacterial H_2_O_2_ scavenging mechanisms and further expands our knowledge of the cold-desert bacteria coping mechanism under extreme environmental stresses.

## Supplementary Information

**Additional file 1: Table S1. **The ability of anti-oxidantstrains to remove various oxidants: differentconcentrations of hydrogen peroxide (0, 1.5, and 3 mM of H_2_O_2_).*Planomicrobium-AX6* is an antioxidantstrain isolated from the Qaidam Basin; the model strain *Escherichia coli* was used as a negative control; *Deinococcus radiodurans* was used as apositive control. **Table S2.** Summary of the sequencing data of strain *Planomicrobium*-AX6. **Table S3** Selected significantly differentially expressed genes (DEGs) for strain*Planomicrobium*-AX6 when exposed for 4hr to H_2_O_2_ concentration (1.5 mM). **Fig. S1. **Response of *Planomicrobium*-AX6to H_2_O_2_ treatments. **A**:Survival rate. On the survival response of exponentially growing *Planomicrobium-*AX6 exposed to H_2_O_2_treatment. **B**: Challenge time. Atspecific time intervals, samples were diluted and plated on agar medium tomonitor cell viability. The data are means of triplicate points. **Fig. S2.** Clusters of Orthologous Groups (COG)classification of the *Planomicrobium*-AX6were annotated and grouped into 21 specific categories.

## Data Availability

Raw sequence data from this study have been submitted to the NCBI sequence read archive under the BioProject accession [PRJNA807914] and are available at the following link: https://www.ncbi.nlm.nih.gov/sra/ PRJNA807914.
