# Supplementary material for: Transcriptional and biochemical analyses of Planomicrobium strain AX6 from Qinghai-Tibetan Plateau, China, reveal hydrogen peroxide scavenging potential
Source: BMC Microbiol. 2022 Nov 5;22:265. doi: 10.1186/s12866-022-02677-w (PMC9636757; doi:10.1186/s12866-022-02677-w)
Supplement: Supplementary file 1 — Additional file 1: Table S1. The ability of anti-oxidantstrains to remove various oxidants: differentconcentrations of hydrogen peroxide (0, 1.5, and 3 mM of H2O2).Planomicrobium-AX6 is an antioxidantstrain isolated from the Qaidam Basin; the model strain Escherichia coli was used as a negative control; Deinococcus radiodurans was used as apositive control. Table S2. Summary of the sequencing data of strain Planomicrobium-AX6. Table S3 Selected significantly differentially expressed genes (DEGs) for strainPlanomicrobium-AX6 when exposed for 4hr to H2O2 concentration (1.5 mM). Fig. S1. Response of Planomicrobium-AX6to H2O2 treatments. A:Survival rate. On the survival response of exponentially growing Planomicrobium-AX6 exposed to H2O2treatment. B: Challenge time. Atspecific time intervals, samples were diluted and plated on agar medium tomonitor cell viability. The data are means of triplicate points. Fig. S2. Clusters of Orthologous Groups (COG)classification of the Planomicrobium-AX6were annotated and grouped into 21 specific categories. [file 12866_2022_2677_MOESM1_ESM.docx]

**Supplementary materials:** *Escherichia coli* was isolated from urine. The growth temperature was 37℃ Medium 1(DSMZ). Medium: NUTRIENT AGAR, Peptone 5.0g Meat extract 3.0g Agar 15g Distilled water 1000ml, Adjust pH to 7.0. For Bacillus strains, the addition of 10.0 mg MnSO4 x H2O is recommended for sporulation. *Deinococcus radiodurans* was isolated from irradiated ground pork and beef. The growth temperature was 30°C Medium 53(DSMZ). Medium 53: CORYNEBACTERIUM AGAR, Casein peptone, tryptic digest 10g, Yeast extract 5g, Glucose 5g, NaCl 5g, Agar 15g, distilled water 1000ml and adjust pH range of 7.2-7.4.

**Table S1** The ability of anti-oxidant strains to remove various oxidants: different concentrations of hydrogen peroxide (0, 1.5, and 3 mM of H_2_O_2_). *Planomicrobium-AX6* is an antioxidant strain isolated from the Qaidam Basin; the model strain *Escherichia coli* was used as a negative control; *Deinococcus radiodurans* was used as a positive control.

|  |  | *Planomicrobium*-AX6 | | |  | *Escherichia coli* | | |  | *Deinococcus radiodurans* | | |
| --- | --- | --- | --- | --- | --- | --- | --- | --- | --- | --- | --- | --- |
| Oxidants |  | 0mM | 1.5mM | 3mM |  | 0mM | 1.5mM | 3mM |  | 0mM | 1.5mM | 3mM |
| DPPH |  | 0.23±0.01d (23.1%) | 0.17±0.01b **(16.6%)** | 0.18±0.02bc (18.4%) |  | 0.06±0.01a (5.7%) | 0.05±0.00a (5.0%) | 0.02±0.01a (2.8%) |  | 0.22±0.00d (22.2%) | 0.21±0.01cd (20.7%) | 0.17±0.01bc (17.3%) |
| O_2_^˙¯^ |  | 0.01±0.00a (1.4%) | 0.18±0.02e **(18.3%)** | 0.09±0.01cd (9.0%) |  | 0.11±0.01d (10.9%) | 0.10±0.00cd (9.5%) | 0.06±0.02bc (6.4%) |  | 0.04±0.00ab (4.0%) | 0.22±0.00f (21.8%) | 0.23±0.00f (22.6%) |
| ^˙^OH |  | 0.05±0.09a (4.5%) | 0.36±0.09bc **(36.4%)** | 0.21±0.07ab (21.2%) |  | 0.11±0.11a (10.6%) | 0.06±0.06a (6.1%) | 0.02±0.02a (1.5%) |  | 0.89±0.04d (89.4%) | 0.53±0.07c (53.0%) | 0.53±0.08c (53.0%) |

2,2-Diphenyl-1-picrylhydrazyl, superoxide, and hydroxyl radical are expressed as DPPH, O_2_^˙¯,^ and ^˙^OH as hydrolysis product H_2_O_2_ (mM). Data are mean values ± deviation from at least three replicates (*P* > 0.05). The number in parentheses is in (mM).

**Table S2** Summary of the sequencing data of strain *Planomicrobium*-AX6

| Sample | Raw reads | Raw bases | Clean reads | Clean bases | Error (%) | Q20 (%) | Q30 (%) | GC (%) | rRNA (%) | Mapped ratio (%) |
| --- | --- | --- | --- | --- | --- | --- | --- | --- | --- | --- |
| AX6 | 22,260,393 | 33,613,193,93 | 21,936,575 | 29,289,679,28 | 0.011 | 98.89 | 96.59 | 44.03 | 32 | 98.90 |
| CK | 23,723,531 | 35,822,531,31 | 23,271,179 | 31,435,409,26 | 0.011 | 98.75 | 96.17 | 45.66 | 48 | 98.62 |

**Table S3** Selected significantly differentially expressed genes (DEGs) for strain *Planomicrobium*-AX6 when exposed for 4 hr to H_2_O_2_ concentration (1.5 mM).

| Functions | Gene ID | Gene name | Annotated function | AX6 | CK | *P*-Value | FDR |
| --- | --- | --- | --- | --- | --- | --- | --- |
| Glutathione metabolism |  |  |  |  |  |  |  |
|  | orf01443 | *ggt* | Hypothetical protein | 148.32 | 56.51 | 0.0043 | 0.0130 |
|  | orf01490 | *PGD*, *gnd* | 6-phosphogluconate dehydrogenase | 58.14 | 14.59 | 0.0054 | 0.0154 |
|  | orf01756 | *PGD*, *gnd* | 6-phosphogluconate dehydrogenase | 56.26 | 10.12 | 0.0006 | 0.0030 |
|  | orf03455 | *E1.11.1.9* | Glutathione peroxidase | 93.78 | 9.24 | 2E-05 | 0.0003 |
| Environmental Bacterial metabolism |  |  |  |  |  |  |  |
|  | orf00449 | *glxK* | MULTISPECIES: glycerate kinase | 45.48 | 133.88 | 0.0022 | 0.0078 |
|  | orf01005 | *E2.3.3.9*, *aceB*, *glcB* | Malate synthase | 2.94 | 29.18 | 0.0202 | 0.0433 |
|  | orf01760 | *kdgK* | 2-dehydro-3-deoxygluconokinase | 8.87 | 41.76 | 0.0041 | 0.0124 |
|  | orf02203 | *glk* | ROK family transcriptional regulator | 31.49 | 123.09 | 0.0004 | 0.0023 |
|  | orf02370 | *E5.1.3.3*, *galM,* | Aldose 1-epimerase | 9.56 | 65.98 | 4E-05 | 0.0005 |
|  | orf03367 | *hemL* | Glutamate-1-semialdehyde aminotransferase | 85.32 | 199.63 | 0.0174 | 0.0382 |
| Thiamine metabolism |  |  |  |  |  |  |  |
|  | orf00071 | *iscS*, *NFS1* | Cysteine desulfurase | 80.44 | 22.14 | 0.0021 | 0.0075 |
|  | orf00072 | *thiI* | Thiamine biosynthesis protein ThiI | 71.03 | 24.84 | 0.0123 | 0.0291 |
|  | orf00452 | *iscS*, *NFS1* | Hypothetical protein | 70.27 | 13.78 | 0.0011 | 0.0047 |
|  | orf00752 | *E2.7.6.2*, *THI80* | Hypothetical protein G159_11040 | 70.67 | 20.09 | 0.012 | 0.0285 |
|  | orf01363 | *E3.1.3.1*, *phoA*, *phoB* | Alkaline phosphatase | 90.47 | 24.04 | 0.0049 | 0.0142 |
|  | orf01438 | *tenA* | Thiaminase | 121.48 | 16.35 | 7.4E-05 | 0.0007 |
|  | orf01439 | *thiM* | Hypothetical protein G159_08755 | 118.9 | 19.71 | 5.9E-05 | 0.0006 |
|  | orf01440 | *thiD* | Hypothetical protein G159_08750 | 92.26 | 10.97 | 1.6E-07 | 1E-05 |
|  | orf01441 | *thiE* | Thiamin-phosphate pyrophosphorylase | 94.31 | 7.64 | 3.1E-08 | 2.9E-06 |
|  | orf02034 | *E3.1.3.1*, *phoA*, *phoB* | Alkaline phosphatase | 88.69 | 26.2 | 0.0006 | 0.0031 |

**Table S3** (Continued)

| Functions | Gene ID | Gene name | Annotated function | AX6^*^ | CK^*^ | *P*-Value | FDR |
| --- | --- | --- | --- | --- | --- | --- | --- |
| Amino acids biosynthesis |  |  |  |  |  |  |  |
|  | orf00599 | *aroB* | Hypothetical protein G159_00580 | 99.38 | 29.18 | 0.0021 | 0.0076 |
|  | orf00600 | *aroC* | Chorismate synthase | 126.92 | 42.26 | 0.0013 | 0.0051 |
|  | orf01449 | *proB* | Glutamate 5-kinase | 60.4 | 7.06 | 0.0003 | 0.0019 |
|  | orf01450 | *proA* | Gamma-glutamyl phosphate reductase | 69.87 | 6.96 | 1.9E-08 | 2.2E-06 |
|  | orf01768 | *serA, PHGDH* | Dehydrogenase | 155.94 | 53.49 | 0.0047 | 0.0139 |
|  | orf02294 | *PGAM, gpmA* | Phosphoglycerate mutase | 360.17 | 22.2 | 6.1E05 | 0.0006 |
|  | orf02703 | *hisIE* | Phosphoribosyl-ATP pyrophosphatase | 142.58 | 47.08 | 0.0088 | 0.0224 |
|  | orf02710 | *hisZ* | ATP phosphoribosyltransferase | 83.48 | 28.68 | 0.0058 | 0.0163 |
| Mismatch repair |  |  |  |  |  |  |  |
|  | orf00091 | *DPO3A1, dnaE* | DNA polymerase III subunit epsilon | 94.61 | 37.56 | 0.0060 | 0.0166 |
|  | orf00673 | *mutS* | DNA mismatch repair protein MutS | 70.86 | 28.24 | 0.0116 | 0.0278 |
|  | orf00707 | *DPO3A2, polC* | DNA polymerase III subunit alpha | 88.2 | 32.08 | 0.0023 | 0.0079 |
|  | orf01237 | *ssb* | Single-stranded DNA-binding protein | 112.13 | 24.63 | 0.0035 | 0.0112 |
|  | orf03010 | *uvrD, pcrA* | Helicase UvrD | 67.25 | 20.77 | 0.0010 | 0.0044 |
|  | orf03475 | *uvrD, pcrA* | Hypothetical protein | 60.07 | 10.46 | 0.0008 | 0.0035 |
| Bacterial chemotaxis |  |  |  |  |  |  |  |
|  | orf02387 | *rbsB* | Sugar ABC transporter, sugar-binding protein | 126.51 | 39.13 | 0.0014 | 0.0055 |
|  | orf02593 | *fliNY, fliN* | Hypothetical protein | 56.91 | 12.48 | 0.0008 | 0.0036 |
|  | orf02594 | *fliM* | Hypothetical protein | 69.59 | 6.98 | 5.08E-06 | 0.0001 |
|  | orf02616 | *motB* | Flagellar motor protein MotB | 103.1 | 16.57 | 1.78E-05 | 0.0003 |
|  | orf02617 | *motA* | Flagellar motor protein MotA | 106.92 | 21.77 | 8.85E-05 | 0.0008 |
| Fatty acid metabolism |  |  |  |  |  |  |  |
|  | orf00333 | *accC* | Acetyl-CoA carboxylase biotin carboxylase | 90.45 | 28.66 | 0.0013 | 0.0051 |
|  | orf00918 | *fabI* | Enoyl-ACP reductase | 99.64 | 19.12 | 0.0002 | 0.0012 |
|  | orf01876 | *accC* | Hypothetical protein | 95.19 | 16.32 | 2.8E-06 | 8.2E-05 |
|  | orf01877 | *accB, bccP* | Acetyl-CoA carboxylase | 324.54 | 43.29 | 0.0229 | 0.0478 |
|  | orf01887 | *fabG* | 3-ketoacyl-ACP reductase | 122.65 | 24.06 | 0.0001 | 0.0009 |

**Table S3** (Continued)

| Functions | Gene ID | Gene name | Annotated function | AX6^*^ | CK^*^ | *P*-Value | FDR |
| --- | --- | --- | --- | --- | --- | --- | --- |
| Cysteine and methionine metabolism |  |  |  |  |  |  |  |
|  | orf00021 | *metK* | S-adenosylmethionine synthetase | 69.89 | 21.57 | 0.0028 | 0.0094 |
|  | orf00147 | *lysC* | Aspartate kinase | 71.75 | 13.52 | 1.4E-06 | 4.9E-05 |
|  | orf00242 | *mtnN, mtn, pfs* | 5'-methylthioadenosine/S-adenosylhomocysteine nucleosidase | 110.87 | 31.57 | 0.0082 | 0.021 |
|  | orf01330 | *yrrT* | SAM-dependent methyltransferase | 75.57 | 5.92 | 2.89E-07 | 1.5E-05 |
|  | orf03215 | *mtnN, mtn, pfs* | Permease | 47.19 | 6.66 | 0.00122 | 0.0050 |
| Pentose phosphate pathway |  |  |  |  |  |  |  |
|  | orf01761 | *eda* | 2-dehydro-3-deoxyphosphogluconate aldolase | 84.5 | 17.82 | 0.0003 | 0.0017 |
|  | orf01776 | *kdgK* | 2-keto-3-deoxygluconate kinase | 101.37 | 20.8 | 0.0075 | 0.0196 |
|  | orf01829 | *E1.1.1.215* | Hypothetical protein G159_04710 | 98.24 | 12.8 | 4.66E-06 | 0.0001 |
|  | orf01982 | *rbsK, RBKS* | Ribokinase | 73.09 | 14.47 | 0.0014 | 0.0055 |
| Two-component system |  |  |  |  |  |  |  |
|  | orf00303 | *pstS* | phosphate-binding protein | 98.48 | 7.23 | 1.06E-07 | 7.9E-06 |
|  | orf01042 | *kinB* | histidine kinase | 50.01 | 11.1 | 0.0007 | 0.0034 |
|  | orf01379 | *dnaA* | chromosomal replication initiation protein | 84.88 | 30.77 | 0.0073 | 0.0192 |
|  | orf01759 | *dctM* | C4-dicarboxylate ABC transporter permease | 47.31 | 7.34 | 7.39E-05 | 0.0007 |
|  | orf01822 | *K11638, citT* | hypothetical protein | 62.55 | 8.84 | 0.0079 | 0.0204 |
|  | orf01825 | *tctB* | Tripartite tricarboxylate transporter | 70.22 | 6.75 | 2.95E-05 | 0.0004 |
|  | orf01826 | *tctA* | Tripartite tricarboxylate transporter | 80.59 | 10.71 | 6.39E-07 | 2.6E-05 |
|  | orf02058 | *malR* | hypothetical protein G159_02325 | 80.06 | 13.71 | 2.76E-05 | 0.0004 |
|  | orf02369 | *atoE* | short-chain fatty acid transporter | 52.91 | 8.68 | 8.56E-05 | 0.0008 |
|  | orf02584 | *fliA* | RNA polymerase sigma factor SigD | 99.1 | 9.43 | 1.92E-08 | 2.2E-06 |
|  | orf02643 | *csrA* | carbon storage regulator | 231.21 | 37.67 | 0.0022 | 0.0078 |

**
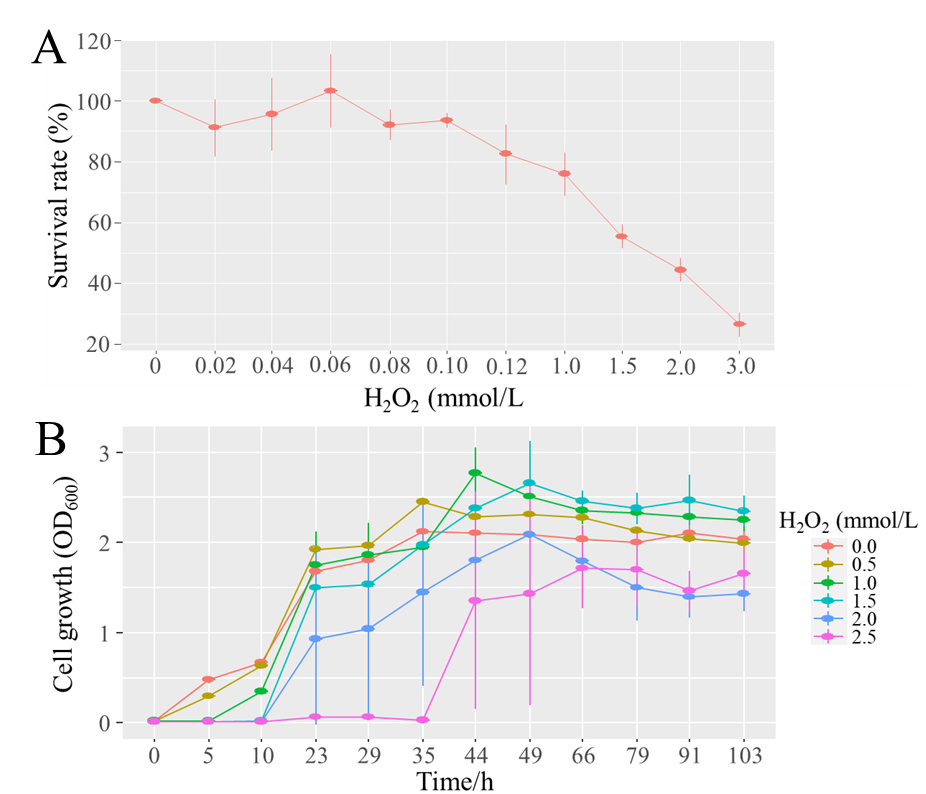
**

**Fig. S1** Response of *Planomicrobium*-AX6 to H_2_O_2_ treatments. **(A)**: Survival rate. On the survival response of exponentially growing *Planomicrobium-*AX6 exposed to H_2_O_2_ treatment. **(B)**: Challenge time. At specific time intervals, samples were diluted and plated on agar medium to monitor cell viability. The data are means of triplicate points.


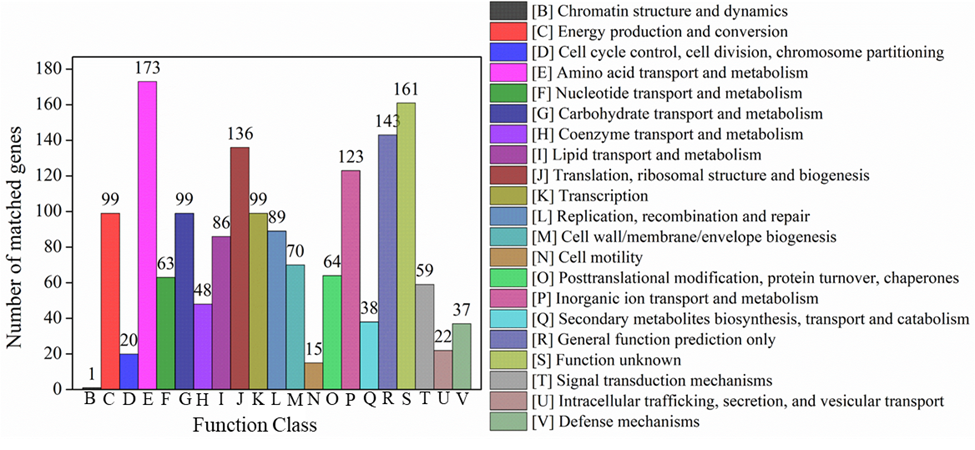


**Fig. S2** Clusters of Orthologous Groups (COG) classification of the *Planomicrobium*-AX6 were annotated and grouped into 21 specific categories.
